# Editorial: Advances in intelligence or nanomedicine-based theranostics for cancers

**DOI:** 10.3389/fonc.2026.1786982

**Published:** 2026-02-25

**Authors:** Han Wang, Jihong Sun, Suhe Wang

**Affiliations:** 1 Department of Radiology, Shanghai General Hospital, Shanghai Jiao Tong University School of Medicine, Shanghai, China; 2 Department of Radiology, Sir Run Run Shaw Hospital, School of Medicine, Zhejiang University, Hangzhou, China; 3 Department of Internal Medicine, Michigan Nanotechnology Institute for Medicine and Biological Sciences, University of Michigan, Ann Arbor, MI, United States

**Keywords:** artificial intelligence, nanomedicine, precision oncology, radiomics, theranostics

## Introduction

Precision oncology has undergone a paradigm shift over the past decade, driven by the convergence of radiomics, radiogenomics, nanoparticle-based molecular imaging, and artificial intelligence (AI). These technologies have redefined cancer theranostics, enabling early detection, personalized treatment stratification, and real-time therapeutic monitoring—capabilities that were once unattainable with conventional approaches. This review synthesizes key findings from a curated Research Topic focused on intelligence- and nanomedicine-based cancer theranostics, highlighting breakthroughs, current challenges, and future directions in this rapidly evolving field.

## Nanomaterials: multimodal tools for imaging and therapy

Iron-based nanomaterials (INMs) have emerged as a cornerstone of nanomedicine-driven oncology, as demonstrated by Xie et al.‘s comprehensive overview. INMs—encompassing iron oxide nanoparticles, element-doped nanocomposites, and metal-organic frameworks—exhibit exceptional versatility in both diagnostic and therapeutic contexts. In imaging, they augment the sensitivity and resolution of magnetic resonance imaging (MRI) and photoacoustic imaging (PAI), while also enabling the development of integrated multimodal imaging platforms that overcome the limitations of single-modality approaches. Therapeutically, INMs are pivotal to advanced strategies such as immunomodulation, magnetic hyperthermia, and synergistic combination therapies, effectively mitigating drug resistance and reducing systemic toxicity. The integration of INMs with AI and radiomics further extends their utility, allowing for precise tumor characterization, treatment optimization, and real-time response assessment. This cross-disciplinary fusion bridges materials science and clinical oncology, laying the groundwork for next-generation theranostic systems that are both targeted and adaptive.

## Photodynamic therapy: synergy as a catalyst for efficacy

Kan et al.‘s systematic review of photodynamic therapy (PDT) underscores the transformative potential of combination therapies in oncology. PDT, when paired with photothermal therapy, radiotherapy, chemotherapy, or immunotherapy, yields a higher therapeutic index and reduced adverse effects compared to monotherapies. The synergistic mechanisms underlying these combinations—such as enhanced immune activation and improved drug delivery—address critical limitations of single-agent treatments, including tumor heterogeneity and drug resistance. These findings reinforce the need for continued research into combination PDT strategies, as they represent a cost-effective and clinically translatable path to improving patient outcomes in both early and late-stage cancer.

## AI and radiomics: data-driven precision in diagnosis

Radiomics and radiogenomics have revolutionized the extraction of quantitative insights from medical imaging, while AI has elevated the translational potential of these approaches. Zhao et al.‘s work on oral squamous cell carcinoma (OSCC) illustrates how AI-driven endoscopy—powered by deep learning models like Mask R-CNN and U-Net—overcomes the limitations of conventional diagnostic methods (e.g., biopsies, CT/MRI) in detecting small or superficial lesions. These AI tools automate lesion detection, segmentation, and classification, reducing operator dependency and human error, particularly in resource-limited settings. However, widespread adoption is hindered by challenges including non-standardized datasets, preprocessing variability, and overfitting. Techniques such as transfer learning and data augmentation are being deployed to address these issues, and ethical considerations (e.g., data privacy) and clinical validation remain paramount. Future efforts must focus on integrating AI seamlessly into clinical workflows to realize its full potential in early OSCC diagnosis and global disease burden reduction.

Wen et al.‘s study on radiation pneumonitis (RP) in breast cancer patients further exemplifies the power of radiomics and machine learning in personalized oncology. By analyzing 1,834 CT-based radiomic features and training eight machine learning classifiers, the researchers identified a robust model (logistic regression) that achieved high accuracy (0.897) and AUC (0.929) for RP diagnosis. The model’s reliance on texture and first-order radiomic features highlights the value of quantitative imaging biomarkers in predicting treatment-related toxicities, enabling proactive intervention and personalized radiotherapy planning.

## Critical research gaps and future directions

Despite the remarkable progress outlined in this Research Topic, several unresolved gaps persist and demand targeted investigation. First, the translational gap between preclinical nanomaterial research and clinical application remains substantial: most INM-based theranostic systems are tested in murine models, and their biocompatibility, pharmacokinetics, and long-term toxicity in human cohorts are poorly characterized. Future work should prioritize large-scale clinical trials to validate the safety and efficacy of INMs, alongside the development of scalable manufacturing protocols to reduce production costs and enable widespread access. Second, AI-driven radiomics and endoscopy lack generalizability across healthcare settings: models trained on data from high-resource institutions often perform poorly on datasets from underserved regions due to variability in imaging equipment and patient populations. Multicenter, cross-geographic data consortia and federated learning approaches— which preserve data privacy while enabling model training across diverse cohorts—are critical to addressing this limitation. Third, the mechanistic understanding of synergistic PDT combinations is incomplete: while empirical evidence confirms their efficacy, the molecular pathways underlying enhanced anti-tumor immunity and drug sensitization require further elucidation. Single-cell sequencing and spatial transcriptomics could unlock these mechanistic insights, guiding the rational design of next-generation PDT combination regimens. Finally, equity in access to precision oncology technologies remains an unmet challenge: AI tools and nanomedicine-based therapies are often unavailable in low- and middle-income countries (LMICs). Future initiatives should focus on developing low-cost, point-of-care AI diagnostic tools and locally manufacturable nanomaterials to ensure these innovations benefit global patient populations.

## Conclusion and future outlook

The four manuscripts in this Research Topic collectively showcase the dynamic frontier of precision oncology, where AI, nanomedicine, and radiomics converge to redefine cancer diagnosis and treatment. While breakthroughs in nanomaterial design, combination therapy, and AI-driven imaging have yielded promising results, critical barriers remain—including clinical validation, ethical compliance, and equitable access to these technologies in underserved regions. Future research must prioritize the translation of preclinical innovations into standardized clinical workflows, and foster collaboration between basic scientists, clinicians, and engineers. As these technologies mature, they hold the potential to shift oncology from a one-size-fits-all paradigm to a truly personalized discipline, where every patient receives tailored theranostic care optimized for their unique tumor biology.

